# Inverse role of distinct subsets and distribution of macrophage in lung cancer prognosis: a meta-analysis

**DOI:** 10.18632/oncotarget.9625

**Published:** 2016-05-26

**Authors:** Pin Wu, Dang Wu, Lufeng Zhao, Lijian Huang, Gang Chen, Gang Shen, Jian Huang, Ying Chai

**Affiliations:** ^1^ Department of Thoracic Surgery, Second Affiliated Hospital, Zhejiang University School of Medicine, Zhejiang University, Hangzhou, 310009, China; ^2^ Department of Radiation Oncology, Second Affiliated Hospital, Zhejiang University School of Medicine, Zhejiang University, Hangzhou, 310009, China; ^3^ Department of Surgical Oncology, Second Affiliated Hospital, Zhejiang University School of Medicine, Zhejiang University, Hangzhou, 310009, China; ^4^ Cancer Institute, Second Affiliated Hospital, Zhejiang University School of Medicine, Zhejiang University, Hangzhou, 310009, China

**Keywords:** tumor-associated macrophage, lung cancer, prognosis, overall survival, meta-analysis

## Abstract

**Background:**

Tumor-associated macrophages (TAMs) play a crucial role in the regulation of local inflammatory and immune response of tumor microenvironment, being associated with worse outcome of several solid tumors. But the prognostic value of tumor-infiltrating TAMs in lung cancer is still controversial.

**Methods:**

We conduct a meta-analysis of 3055 patients in 21 studies searched from PubMed and Medline to investigate the correlation between tumor-infiltrating TAMs, including distinct TAM subsets and tissue distribution, and survival of lung cancer. Survival data were computed into odds ratios (ORs) and pooled using Mantel–Haenszel random-effect model. All statistical tests were two-sided.

**Results:**

High density of tumor-infiltrating TAMs was significantly associated with worse overall survival (OS) at 3 years (OR = 2.45, 95% CI = 1.25 to 4.80, P = 0.009) and 5 years (OR = 2.04, 95% CI = 1.03 to 4.01, P = 0.04) of lung cancer. Results for disease free survival (DFS) were similar. M2 subset was associated with worse 3 year-OS and 5 year-OS, whereas M1 subset was associated with better 3-year OS and 5-year OS. Elevated TAM density in tumor stroma was associated with worse OS at 3 years and 5 years, while elevated TAMs in tumor islet/tumor stroma were associated with better OS at 3 years and 5 years.

**Conclusions:**

Increased tumor-infiltrating TAMs are associated with poor prognosis of lung cancer. M2 subset and TAMs in tumor stroma were associated with worse survival, while M1 subset and TAMs in tumor islet were associated with favorable survival of lung cancer.

## INTRODUCTION

Tumor-associated macrophages (TAMs) play a crucial role in the local inflammatory response and immunosurveillance of cancer [1, 2]. Plentiful studies have demonstrated that tumor-infiltrating TAMs are associated with worse outcome of human lung cancer [3–10]. However, other studies reported that tumor-infiltrating TAMs were associated with favorable outcome of human lung cancer [11, 12]. It is reported that the type, functional orientation, density, and location of immune cells within distinct tumor regions are all associated with cancer patient survival [13]. Therefore, the divergence maybe due to both the heterogeneity of TAM subsets and functional plasticity of TAMs in local tumor microenvironment [14, 15].

TAMs are generally characterized by the expression of cell surface marker CD68. However, the results from studies evaluating the correlation between TAMs and survival of lung cancer using CD68 as a marker of TAMs are still contradictory [5, 7, 9, 10, 16–20]. Recently, accumulating studies subdivided TAMs into the classically activated M1 phenotype and the alternatively activated M2 phenotype with an opposite function in tumor immunity [21]. It is reported that tumor-infiltrating M1 is associated with favorable outcome of human lung cancer [11]. On the contrary, some studies suggested that tumor-infiltrating M2 was associated with worse outcome of human lung cancer [8, 9, 11, 12, 18, 22]. Moreover, TAMs in tumor nest were reported to be associated with better outcome of human lung cancer [5, 11, 16–18, 20]. Other studies showed that increased TAMs in tumor stroma were negatively correlated with the survival of lung cancer [5, 11, 16–20, 22].

Thus, further studies are needed to clarify the role of tumor-infiltrating TAMs, subsets and intratumoral distribution of TAMs in prognostic prediction of lung cancer. We therefore conducted an exhaustive meta-analysis combining evidence to evaluate the prognostic value of TAMs in human lung cancer. This meta-analysis came into a conclusion that elevated TAMs were associated with worse survival of lung cancer, especially M2 phenotype and TAMs distributing in tumor stroma. While elevated M1 phenotype and TAMs distributing in tumor islet were associated with better survival of lung cancer. Our study suggests that the subsets and tissue distribution of TAMs are very meaningful in prognostic prediction of lung cancer.

## RESULTS

### Search results and study characteristics

Literature searches yield 2338 records and the results are shown in Supplementary Figure S1. The potentially relevant articles were screened for eligibility by duplication, language, abstract and article type, and 1436 records were excluded. Next, 692 citations were excluded for detailed evaluation and at last 21 studies with survival data were included. Characteristics of studies including OS or DFS data are shown in Table 1. A total of 3055 patients were included in those studies.

**Table 1:** Characteristics of studies included in the meta-analysis

Ref	Patient No.	Age, median (range)	Male/Female	Stage	Follow-up, median (range)	Marker	Tissue Distribution	Cutoff value	Antibody (Clone)	NOS Score
**Studies including OS**										
Carus, A., et al. (2013)	335	≥65, 200; <65, 135	194/141	I–IIIA	NR	CD163	Tumor islet	≥0.21% of tissue area	Anti-CD163(EDHu-1)	7
							Tumor stroma	≥2.19% of tissue area		
Chen, J. J., et al. (2003)	35	60.3	24/11	I–IIIA	NR	CD68	Tumor islet and stroma	≥162 cells/field (200×)	Anti-CD68(NR)	7
Dai, Fuqiang., et al. (2010)	99	60 (37 – 80)	80/19	I-IV	96	CD68	Tumor islet and stroma	≥15 cells/field (400×)	Anti-CD68(KP1)	8
							Tumor islet			
							Tumor stroma			
							Tumor islet/stroma			
Hirayama, S., et al. (2012)	208	69 (46–88)	188/20	I-IIIA	68.4	CD204	Tumor stroma	≥30 cells/field(400×)	Anti-CD204(A-E5)	8
							Tumor islet	≥9 cells/field (400×)		
Ho, C. C., et al. (2008)	68	NR	40/28	I-III	41	TREM-1	Tumor islet and stroma	≥15 cells/field (400×)	Anti-TREM-1(AF1278)	7
Kawai, O., et al. (2008)	199	62 (39-79)	139/60	IV	NR	CD68	Tumor islet/stroma	≥1	Anti-CD68(NR)	7
							Tumor islet	≥13 cells/field (400×)		
							Tumor stroma	≥12 cells/field (400×)		
Kim, D. W., et al. (2008)	144	60.4	106/38	IA-IV	NR	CD68	Tumor stroma	≥233 cells/mm^2^ (400×)	Anti-CD68(M0876)	7
							Tumor islet	≥28 cells/mm^2^ (400×)		
Li, Y., et al. (2015)	159	61 (44-77)	109/50	I-III	46 (2-120)	CD68	Tumor islet and stroma	NR	Anti-CD68(ED1)	8
						Osteopontin/CD68	Tumor islet and stroma	NR	Anti-OPN(NR); Anti-CD68(KP1)	
Ma, J., et al. (2010)	100	NR	81/19	I-IV	Max 96	CD68/HLA-DR	Tumor islet and stroma	NR	Anti-CD68(KP1); Anti-HLA-DR(LN3)	7
							Tumor islet	NR		
							Tumor stroma	NR		
						CD68/CD163	Tumor islet and stroma	NR	Anti-CD68(KP1); Anti-CD163(10D6)	
							Tumor islet	NR		
							Tumor stroma	NR		
Ohtaki, Y., et al. (2010)	170	62 (33–85)	85/85	IA-IIIA	121.2	CD68	Tumor islet	25 cells/mm2 (400×)	Anti-CD68(NR)	8
						CD204	Tumor stroma	15 cells/mm2 (400×)	Anti-CD204(A-E5)	
Pei, B.-x., et al. (2014)	417	NR	231/186	I-III	43 (2-120)	CD68	Tumor stroma	positive of CD68+ cells	Anti-CD68(KP1)	7
Takahashi, A., et al. (2013)	115	68 (22-86)	98/17	IA-IV	52.8	CD204	Tumor islet and stroma	≥20 cells/field (400×)	Anti-CD204(A-E5)	8
Takanami, I., et al. (1999)	113	62 (30-79)	66/47	I-IV	NR	CD68	Tumor islet and stroma	Densities> 32	Anti-CD68(KP1)	7
Welsh, T. J., et al. (2005)	162	NR	NR	I-IV	NR	CD68	Tumor islet	≥131 cells/mm^2^ (200×)	Anti-CD68(PGM1)	6
							Tumor stroma	≥174 cells/mm^2^ (200×)		
							Tumor islet/stroma	≥Median value		
Zeni, E., et al. (2007)	47	63.9	43/7	I-IV	NR	IL-10/CD68	Tumor islet and stroma	≥16.3% of tissue area	Anti-IL-10(NR); Anti-CD68(M0814)	7
Zhang, B., et al. (2011)	65	NR	38/27	I-IV	NR	CD68	Tumor islet and stroma	≥102 cells/field (100×)	Anti-CD68(NR)	6
						CD68/CD206	Tumor islet and stroma	≥82 cells/field (100×)		
Zhang, W., et al. (2013)	67	71(45-85)	22/27	I-III	53.3 (2.1–201.7)	CD68	Tumor islet and stroma	≥50% of tissue area	Anti-CD68(514H12)	8
**Studies including DFS**										
Chen, J. J., et al. (2003)	35	60.3	24/11	I–IIIA	NR	CD68	Tumor islet and stroma	≥162 cells/field (200×)	Anti-CD68(NR)	7
Hirayama, S., et al. (2012)	208	69 (46–88)	188/20	I-IIIA	68.4	CD204	Tumor stroma	≥30 cells/field(400×)	Anti-CD204(A-E5)	8
							Tumor islet	≥9 cells/field (400×)		
Ho, C. C., et al. (2008)	68	NR	40/28	I-III	41	TREM-1	Tumor islet and stroma	≥15 cells/field (400×)	Anti-TREM-1(AF1278)	7
Ito, M., et al. (2012)	304	NR	139/165	I	87(5-181)	CD204	Tumor islet and stroma	≥8 cells/field (400×)	Anti-CD204(A-E5)	7
Kaseda, K., et al. (2013)	41	NR	NR	I	NR	CD204	Tumor stroma	NR	Anti-CD204(A-E5)	6
Li, Y., et al. (2015)	159	61 (44-77)	109/50	I-III	46 (2-120)	CD68	Tumor islet and stroma	NR	Anti-CD68(ED1)	8
						Osteopontin/CD68	Tumor islet and stroma	NR	Anti-OPN(NR); Anti-CD68(KP1)	
Maeda, R., et al. (2014)	207	NR	94/113	I	NR	CD204	Tumor islet and stroma	≥8 cells/field (400×)	Anti-CD204(A-E5)	6
Pei, B.-x., et al. (2014)	417	NR	231/186	I-III	43 (2-120)	CD68	Tumor stroma	positive of CD68+ cells	Anti-CD68(KP1)	7
Takahashi, A., et al. (2013)	115	68 (22-86)	98/17	IA-IV	52.8	CD204	Tumor islet and stroma	≥20 cells/field (400×)	Anti-CD204(A-E5)	8

NR: Not Reported; DFS: disease-free survival; OS: overall survival.

### Evaluation and density of TAM

A description of the antibodies, detection and definition method of TAM density used in the included studies is shown in Table 1. Various markers were used for the evaluation of TAM density. Seven studies used CD68 antibody, three studies used CD204, one study used CD68 and CD206, one study used CD68 and HLA-DR or CD68 and CD163, one study used TREM-1, one study used IL-10 and CD68 and one study used osteopontin and CD68.

### Association of TAM with survival of lung cancer

**Figure 1:**
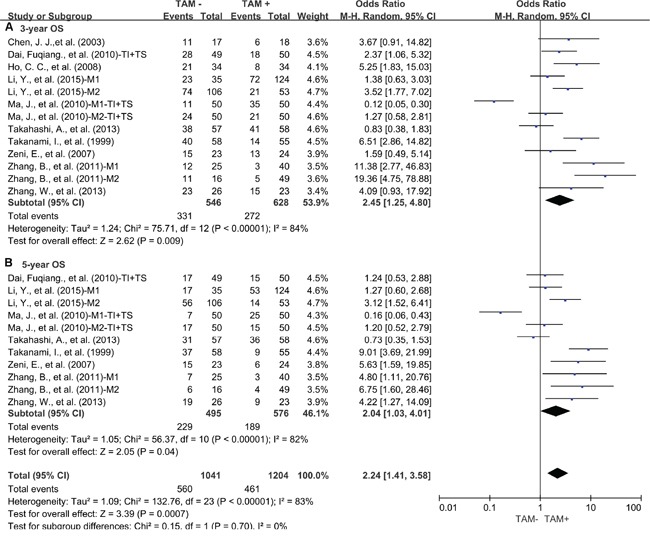
Forest plots showing odds ratios of high density of TAMs versus low density of TAMs for overall survival (OS) at 3 and 5 years **A.** 3-year OS; **B.** 5-year OS. TI: tumor islet; TS: tumor stroma; M1: marker1; M2: marker2.

**Figure 2:**
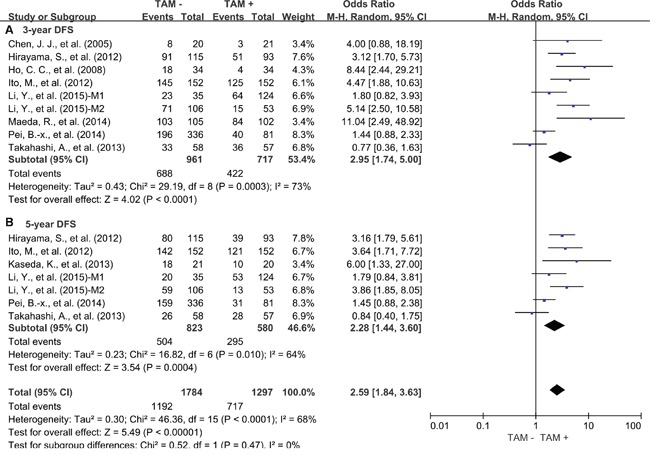
Forest plots showing odds ratios of high density of TAMs versus low density of TAMs for disease free survival (DFS) at 3 and 5 years **A.** 3-year DFS; **B.** 5-year DFS. M1: marker1; M2: marker2.

Our study showed that elevated density of tumor-infiltrating TAMs was associated with worse 3-year OS (OR = 2.45, 95% confidence interval (CI) = 1.25 to 4.80, P = 0.009) (Figure 1A) and 5-year OS (OR = 2.04, 95% CI = 1.03 to 4.01, P = 0.04) (Figure 1B) of lung cancer. Regarding to DFS, high density of tumor-infiltrating TAMs was also associated with worse 3-year DFS (OR = 2.95, 95% CI = 1.74 to 5.00, P < 0.0001) (Figure 2A) and 5 year DFS (OR = 2.28, 95% CI = 1.44 to 3.60, P = 0.0004) (Figure 2B) of lung cancer.

**Figure 3:**
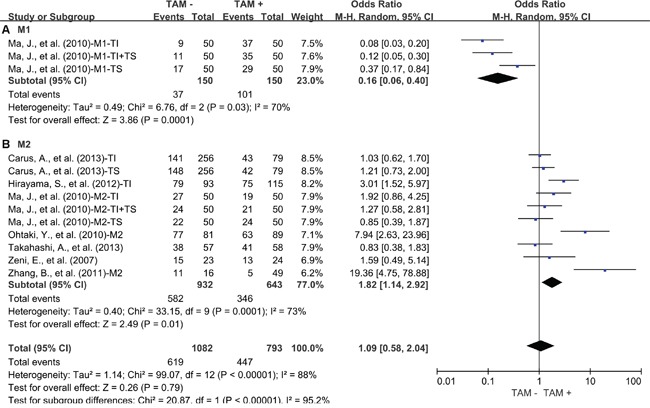
Subgroup analysis of 3-year OS by high density of different TAM subsets **A.** M1; **B.** M2. TI: tumor islet; TS: tumor stroma; M1: CD68 and HLA-DR positive cells; M2: CD163 positive cells, CD204 positive cells, CD68 and CD163 positive cells, CD68 and CD206 positive cells or IL-10 and CD68 positive cells.

**Figure 4:**
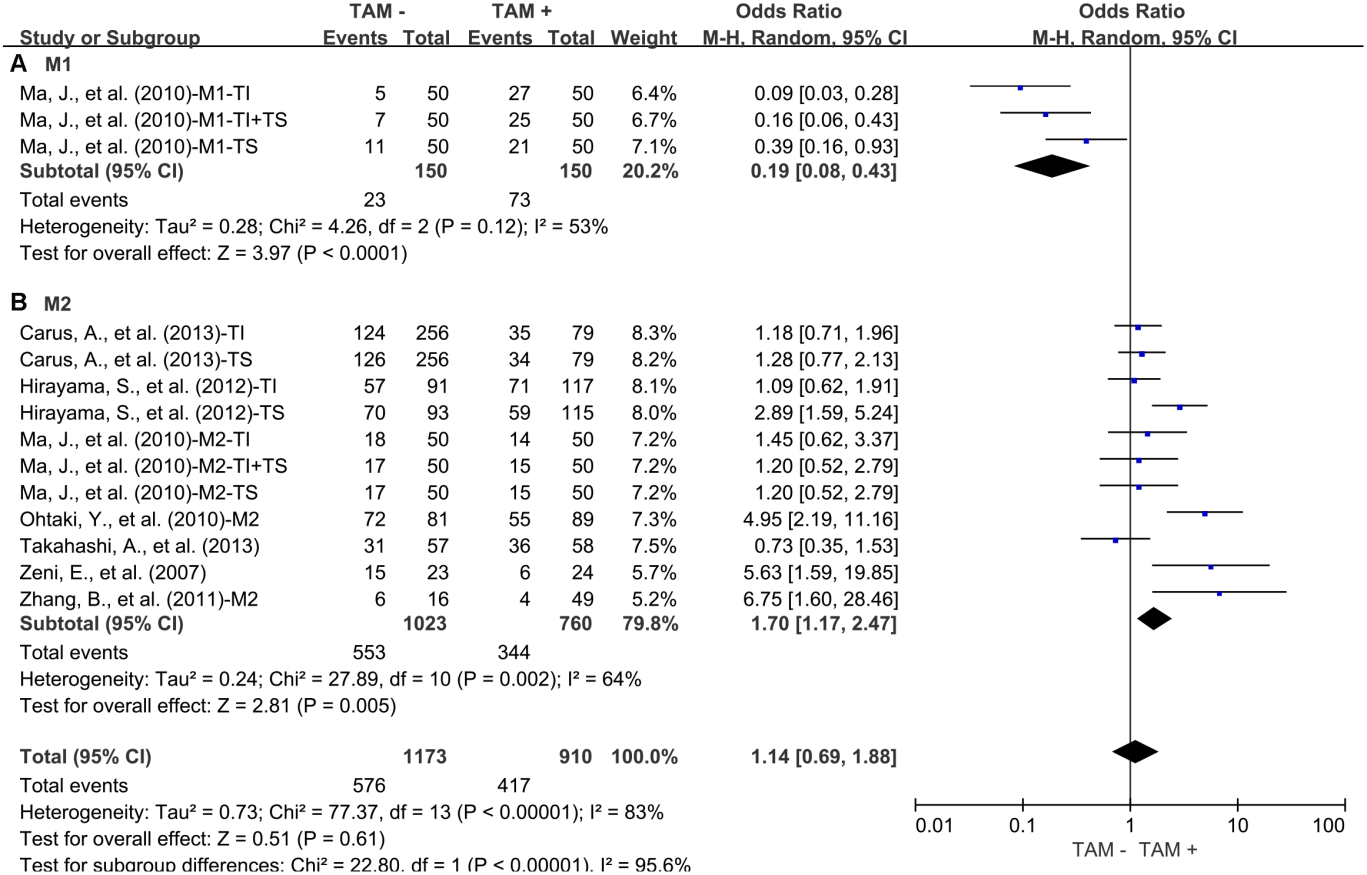
Subgroup analysis of 5-year OS by high density of different TAM subsets **A.** M1; **B.** M2. TI: tumor islet; TS: tumor stroma; M1: CD68 and HLA-DR positive cells; M2: CD163 positive cells, CD204 positive cells, CD68 and CD163 positive cells, CD68 and CD206 positive cells or IL-10 and CD68 positive cells.

Regarding to the subsets of TAMs, subgroup meta-analysis showed that elevated density of tumor-infiltrating M1 was associated with favorable 3-year OS of lung cancer (OR = 0.16, 95% CI = 0.06 to 0.40, P = 0.0001) (Figure 3A). In contrast, elevated density of tumor-infiltrating M2 was associated with worse 3-year OS of lung cancer (OR = 1.82, 95% CI = 1.14 to 2.92, P = 0.01) (Figure 3B). Similar to the results of 3-year OS, elevated density of tumor-infiltrating M1 was associated with favorable 5-year OS of lung cancer (OR = 0.19, 95% CI = 0.08 to 0.43, P < 0.0001) (Figure 4A). In contrast, elevated density of tumor-infiltrating M2 was associated with worse 5-year OS of lung cancer (OR = 1.70, 95% CI = 1.17 to 2.47, P = 0.002) (Figure 4B). Further study showed that neither M2 in tumor islet nor tumor stroma was associated with 3-year OS of lung cancer (Supplementary Figure S2A). However, M2 in tumor stroma was associated with worse 5-year OS of lung cancer (OR = 2.13, 95% CI = 1.13 to 4.00, P = 0.01) (Supplementary Figure S2B) but not M2 in tumor islet.

**Figure 5:**
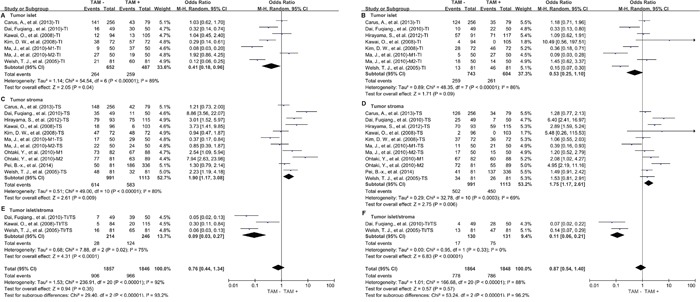
Subgroup analysis of OS by high density of TAMs in different tumor distribution **A.** High density of TAMs in TI and 3-year OS; **B.** High density of TAMs in TI and 5-year OS; **C.** High density of TAMs in TS and 3-year OS; **D.** High density of TAMs in TS and 5-year OS; **E.** High value of TAMs in TI/TS and 3-year OS; **F.** High value of TAMs in TI/TS and 5-year OS. TI: tumor islet; TS: tumor stroma; M1: marker1; M2: marker2.

It is interesting that high density of TAMs in tumor islet was correlated with favorable 3-year OS of lung cancer (OR = 0.41, 95% CI = 0.18 to 0.96, P = 0.04) (Figure 5A), but not 5-year OS of lung cancer (Figure 5B). In contrast, elevated density of TAMs in tumor stroma was associated with worse 3-year OS (OR = 1.90, 95% CI = 1.17 to 3.08, P = 0.009) (Figure 5C) and 5-year OS (OR = 1.75, 95% CI = 1.17 to 2.61, P = 0.006) (Figure 5D) of lung cancer. However, high value of tumor islet/tumor stroma rate was significantly correlated with favorable 3-year OS (OR = 0.09, 95% CI = 0.03 to 0.27, P < 0.0001) (Figure 5E) and 5-year OS (OR = 0.11, 95% CI = 0.06 to 0.21, P < 0.00001) (Figure 5F) of lung cancer.

We also elicit a subgroup meta-analysis according to tumor stage. Results showed that elevated density of tumor-infiltrating TAM was associated with worse 3-year OS (Supplementary Figure S3A) and 5-year OS (Supplementary Figure S3B) in stage I-III.

### Sensitivity analyses

Removal of the studies that used a non-classical marker of TAM (TREM-1 and CD68 combine with IL-10) did not substantially affect the association between TAM density and worse 3- or 5-year OS (OR = 2.31, 95% CI = 1.13 to 4.68, p = 0.02; OR = 2.29, 95% CI = 1.10 to 4.74, p = 0.03, respectively). Exclusion of these studies did not reduce heterogeneity for 3- or 5-year OS (Cochran's Q P < 0.00001, I^2^ = 85%; Cochran's Q P < 0.00001, I^2^ = 82%, respectively). Meta-regression analysis showed that publication year, country, gender and NOS score did not contribute to the heterogeneity (data not shown).

### Publication bias

Funnel plot analysis and Egger's test showed that there was no statistical evidence of publication bias in our meta-analysis.

## DISCUSSION

Numerous studies have demonstrated that TAMs promote cancer initiation and progression via inducing angiogenesis, enhancing tumor cell migration and invasion, and suppressing antitumor immunity [2]. However, the correlation between TAMs and outcome of lung cancer is still under debate. Our comprehensive meta-analysis of 3055 patients included in 21 different studies demonstrates that elevated density of TAMs in tumor is correlated with poor prognosis of lung cancer. However, the correlation between TAMs and outcome of lung cancer is distinct between TAM subsets and intratumoral distribution of TAM. Elevated M2 and TAM in tumor stroma is correlated with poor outcome of lung cancer. In contrast, increased M1 and TAM in tumor nest is correlated with favorable outcome of lung cancer. These findings suggest both the intratumoral distribution and subsets of TAMs are important factors affecting the prognosis of human lung cancer.

Macrophages have traditionally been divided into M1 and M2 subsets and are distinguished by their cell surface markers [15, 23]. Generally, M1 is suggested to play a crucial role in killing intracellular pathogens and suppressing tumor progression, while M2 can facilitate tumor development in multiple mechanisms [24]. In line with experimental research, our study demonstrates that elevated M1 is correlated with better OS of lung cancer. In contrast, elevated M2 is correlated with worse OS of lung cancer. Further analysis shows that only elevated M2 in tumor stroma is correlated with worse OS of human lung cancer but not in tumor islet. These findings indicate that polarized state of TAM in tumor microenvironment is correlated with clinical outcome of lung cancer patient, and approaches to reprogram TAMs from an M2 to an M1-like phenotype are promising in lung cancer therapy.

Accumulated studies reported that there is an inverse association between TAM and prognosis of human lung cancer in tumor islet and tumor stroma [5, 11, 16, 17, 20, 25]. Our study shows that elevated CD68+ TAMs in tumor islet are correlated with better OS of lung cancer. In contrast, elevated CD68+ TAMs in tumor stroma are correlated with worse OS of lung cancer. It is interesting that there is a prominent correlation between the ratio of TAMs in tumor islet/tumor stroma and favorable outcome of human lung cancer [5, 16, 20]. However, the correlation between intratumoral distribution of TAM and progress of other human solid tumors are still unclear. Moreover, the discrepant role and underling regulatory mechanisms of TAMs in the different interspace of tumor microenvironment, such as tumor islet and tumor stroma, are needed to be further studied.

This study has several important implications. First, it shows that high TAM density is associated with poor outcome of lung cancer, which suggests that TAMs may be a potential therapeutic target. Second, it suggests the tissue distribution of TAMs plays an important role in tumor progression and prognostic prediction of human lung cancer. Third, it highlights the distinct role of M1 and M2 subsets in tumor progression and prognostic prediction of human lung cancer, which suggests that the key factors involved in M2 polarization may also be a potential therapeutic target.

Some limitations also exist in this meta-analysis. First, the markers and cut-off values for assessing TAMs expression are inconsistent. Second, significant heterogeneity observed among studies cannot be completely interpreted despite the use of appropriate meta-analytic technique with random-effect models. Finally, small studies with negative results may not be published, resulting in publication bias.

In conclusion, our analyses show that elevated density of TAMs in human lung cancer tissues, especially M2 or CD68+ TAMs in tumor stroma, is associated with worse prognosis in human lung cancer, which suggests that directly targeting TAMs or M2, or reprogramming TAMs from an M2 to an M1phenotype could be promising therapeutic approaches for lung cancer.

## MATERIALS AND METHODS

This meta-analysis was carried out in accordance with preferred reporting items for systematic reviews and meta-analyses statement [26].

### Identification and selection of studies

PubMed and Medline were searched for studies evaluating the density of tumor-infiltrating TAM and survival in lung cancer from 1964 to August 2015. The search terms included “Macrophages” and “Lung Neoplasms” and the results were limited to human studies of lung cancer. We identified a total of 1172 and 1166 entries, respectively. Eligibility criteria were the measurement of tumor-infiltrating TAM by immunohistochemistry (IHC), availability of survival data for at least 3-year survival, and publication in English. Studies Citation lists of retrieved articles were manually screened to ensure sensitivity of the search strategy. Study selection was based on the association of the density of TAM in tumor tissue and survival. Inter-reviewer agreement was assessed using Cohen's kappa coefficient. Disagreement was resolved by consensus.

### Endpoints of interest

Overall survival (OS) or disease free survival (DFS) at 3 and 5 years were the primary endpoints. Tumors were classified by TAM density using cut-off as defined by individual studies.

### Data collection process

Two authors (Pin Wu and Dang Wu) independently extracted information using predefined data abstraction forms. The following details were extracted by 2 reviewers (Pin Wu and Dang Wu): number of patients, antibody used for the evaluation, technique used to quantify TAM, and cut-off to determine high density of TAM. Survival data were extracted from tables or Kaplan–Meier curves for both TAM low (control group) and high group (experimental group). The studies included in our meta-analysis were all cohort studies. Two independent authors evaluated the quality of each included study using Newcastle-Ottawa Scale (NOS) [27]. The studies with 6 scores or more were considered as high quality studies. A consensus NOS score for each item was achieved finally.

### Data synthesis

The relative frequency of survival at 3 and 5 years between the control and experimental groups was expressed as an odds ratio (OR) and its 95% confidence interval (CI). Sensitivity analyses were carried out for different analytical methods and cut-offs for defining the density of TAM and NOS scores for quality assessment of included studies.

### Statistical analysis

Data were extracted from the primary publications and combined into a meta-analysis using RevMan 5.3 analysis software (Cochrane Collaboration, Copenhagen, Denmark). Estimates of ORs were weighted and pooled using the Mantel–Haenszel random effect model. Statistical heterogeneity was assessed using the Cochran's Q and I^2^ statistics. Differences between subgroups were assessed using methods as previous described by Deeks et al. [28]. Meta-regression analysis was conducted using Stata 12.0 software (StataCorp LP, College Station, TX). All statistical tests were two-sided, and statistical significance was defined as *P* value less than 0.05. No correction was made for multiple statistical testing.

## SUPPLEMENTARY FIGURES


