# Supplementary material for: Inverse role of distinct subsets and distribution of macrophage in lung cancer prognosis: a meta-analysis
Source: Oncotarget. 2016 May 26;7(26):40451–60. doi: 10.18632/oncotarget.9625 (PMC5130019; doi:10.18632/oncotarget.9625)
Supplement: Supplementary file 1 [file oncotarget-07-40451-s001.pdf]

Inverse role of distinct subsets and distribution of macrophage in lung cancer prognosis: a meta-analysis

SUPPLEMENTARY FIGURES

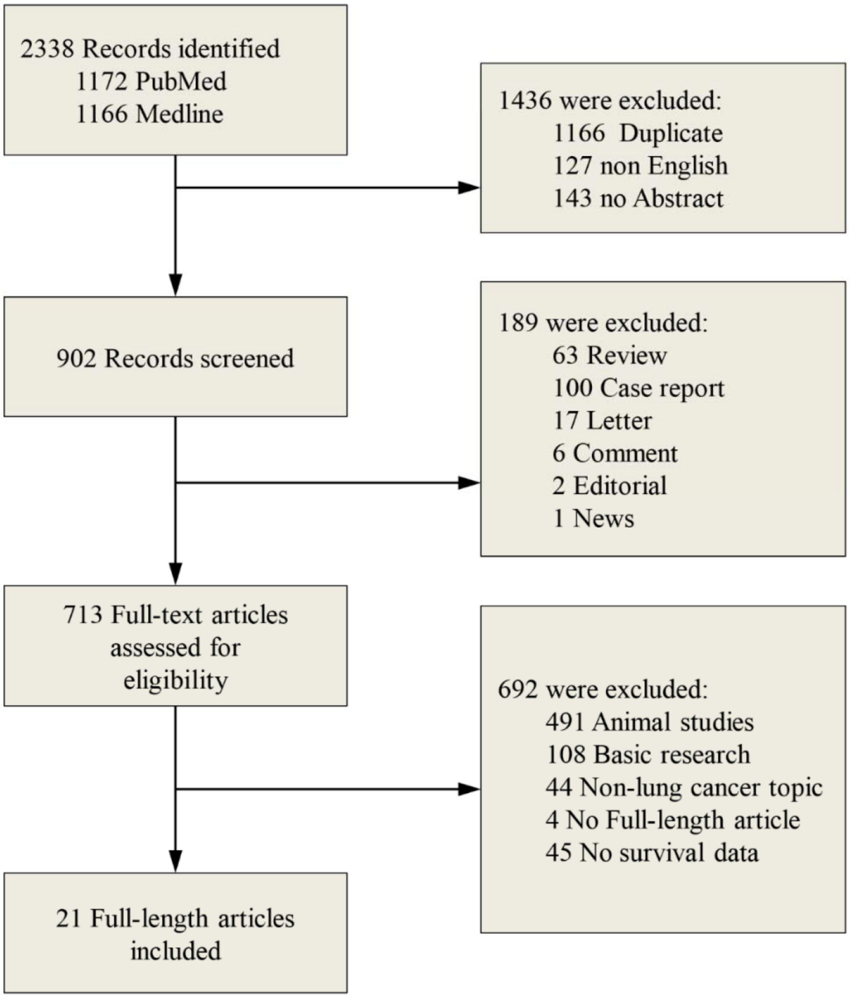

Supplementary Figure S1: Flow diagram of study selection.

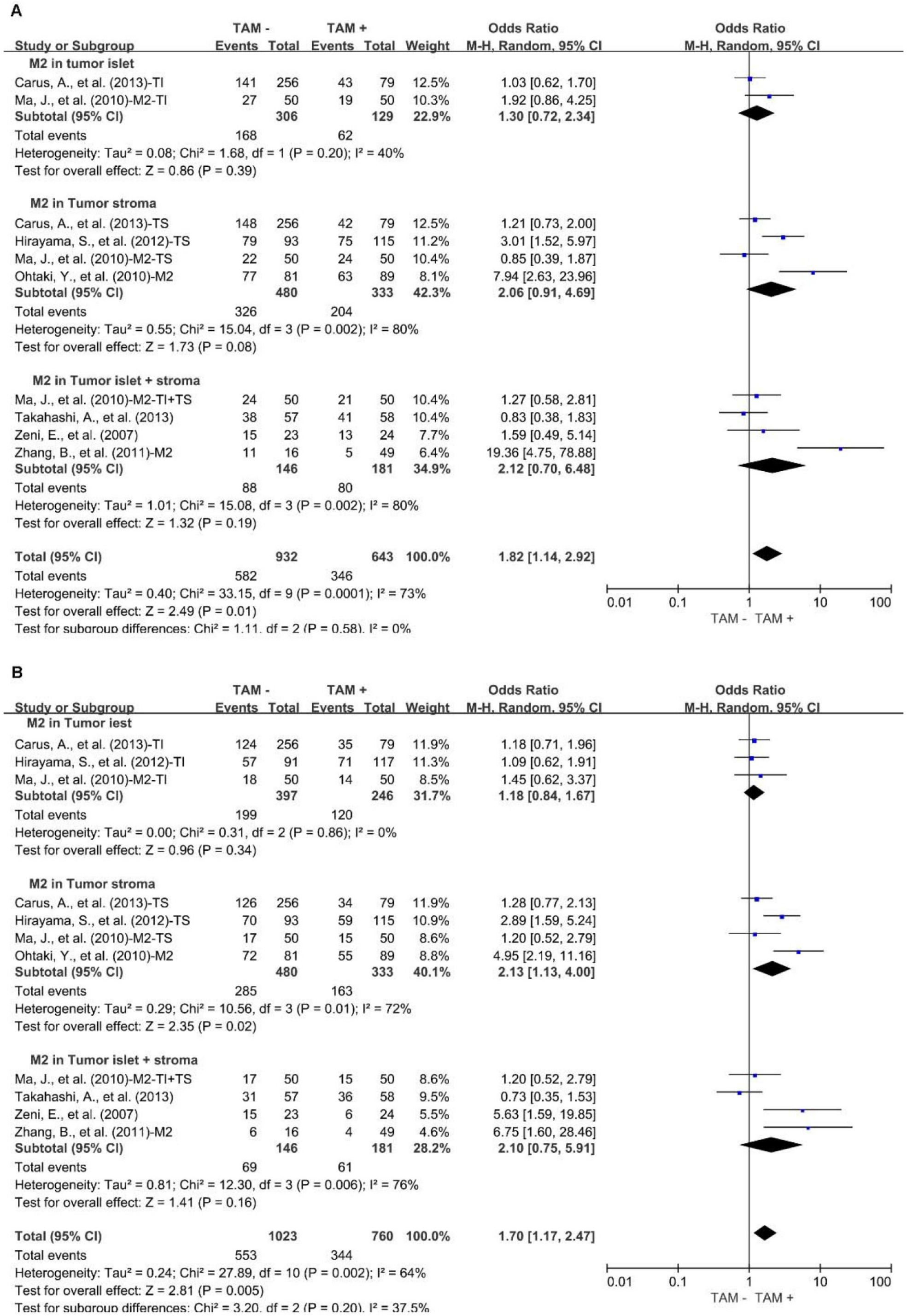

Supplementary Figure S2: Subgroup analysis of overall survival (OS) by high density of M2 subset in different tumor distribution. A. 3-year OS; **B.** 5-year OS. TI: tumor islet; TS: tumor stroma; M1: CD68 and HLA-DR positive cells; M2: CD163 positive cells, CD204 positive cells, CD68 and CD163 positive cells, CD68 and CD206 positive cells or IL-10 and CD68 positive cells.

A

Study or Subgroup	TAM - Events	TAM - Total	TAM + Events	TAM + Total	Weight	Odds Ratio M-H, Random, 95% CI
**I-III**						
Carus, A., et al. (2013)-TI	141	256	43	79	9.3%	1.03 [0.62, 1.70]
Carus, A., et al. (2013)-TS	148	256	42	79	9.3%	1.21 [0.73, 2.00]
Chen, J. J.,et al. (2003)	11	17	6	18	4.6%	3.67 [0.91, 14.82]
Hirayama, S., et al. (2012)-TS	79	93	75	115	8.2%	3.01 [1.52, 5.97]
Ho, C. C., et al. (2008)	21	34	8	34	6.1%	5.25 [1.83, 15.03]
Li, Y., et al. (2015)-M1	23	35	72	124	7.6%	1.38 [0.63, 3.03]
Li, Y., et al. (2015)-M2	74	106	21	53	8.2%	3.52 [1.77, 7.02]
Ohtaki, Y., et al. (2010)-M1	73	82	67	88	7.2%	2.54 [1.09, 5.94]
Ohtaki, Y., et al. (2010)-M2	77	81	63	89	5.8%	7.94 [2.63, 23.96]
Pei, B.-x., et al. (2014)	50	81	186	336	9.3%	1.30 [0.79, 2.14]
Zhang, W., et al. (2013)	23	26	15	23	4.3%	4.09 [0.93, 17.92]
**Subtotal (95% CI)**		**1067**		**1038**	**79.9%**	**2.26 [1.53, 3.34]**
Total events	720		598			
Heterogeneity: Tau² = 0.26; Chi² = 28.77, df = 10 (P = 0.001); I² = 65%						
Test for overall effect: Z = 4.10 (P < 0.0001)						
**IV**						
Kawai, O., et al. (2008)-TI	12	94	13	105	7.3%	1.04 [0.45, 2.40]
Kawai, O., et al. (2008)-TI/TS	5	84	20	115	6.2%	0.30 [0.11, 0.84]
Kawai, O., et al. (2008)-TS	18	96	6	103	6.5%	3.73 [1.41, 9.85]
**Subtotal (95% CI)**		**274**		**323**	**20.1%**	**1.06 [0.28, 4.08]**
Total events	35		39			
Heterogeneity: Tau² = 1.19; Chi² = 12.26, df = 2 (P = 0.002); I² = 84%						
Test for overall effect: Z = 0.08 (P = 0.93)						
**Total (95% CI)**		**1341**		**1361**	**100.0%**	**1.98 [1.34, 2.93]**
Total events	755		637			
Heterogeneity: Tau² = 0.36; Chi² = 44.05, df = 13 (P < 0.0001); I² = 70%						
Test for overall effect: Z = 3.45 (P = 0.0006)						
Test for subgroup differences: Chi² = 1.12, df = 1 (P = 0.29), I² = 10.9%						

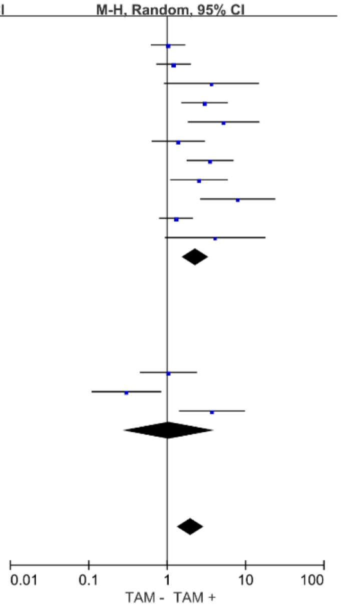

B

Study or Subgroup	TAM - Events	TAM - Total	TAM + Events	TAM + Total	Weight	Odds Ratio M-H, Random, 95% CI
**I-III**						
Carus, A., et al. (2013)-TI	124	256	35	79	12.0%	1.18 [0.71, 1.96]
Carus, A., et al. (2013)-TS	126	256	34	79	12.0%	1.28 [0.77, 2.13]
Hirayama, S., et al. (2012)-TI	57	91	71	117	11.2%	1.09 [0.62, 1.91]
Hirayama, S., et al. (2012)-TS	70	93	59	115	10.7%	2.89 [1.59, 5.24]
Li, Y., et al. (2015)-M1	17	35	53	124	8.7%	1.27 [0.60, 2.68]
Li, Y., et al. (2015)-M2	56	106	14	53	9.1%	3.12 [1.52, 6.41]
Ohtaki, Y., et al. (2010)-M1	67	82	60	88	9.1%	2.08 [1.02, 4.27]
Ohtaki, Y., et al. (2010)-M2	72	81	55	89	8.0%	4.95 [2.19, 11.16]
Pei, B.-x., et al. (2014)	41	81	137	336	12.4%	1.49 [0.91, 2.42]
Zhang, W., et al. (2013)	19	26	9	23	4.8%	4.22 [1.27, 14.09]
**Subtotal (95% CI)**		**1107**		**1103**	**98.0%**	**1.86 [1.36, 2.55]**
Total events	649		527			
Heterogeneity: Tau² = 0.14; Chi² = 21.35, df = 9 (P = 0.01); I² = 58%						
Test for overall effect: Z = 3.89 (P = 0.0001)						
**IV**						
Kawai, O., et al. (2008)-TI	4	94	0	105	1.0%	10.49 [0.56, 197.51]
Kawai, O., et al. (2008)-TS	2	96	0	103	1.0%	5.48 [0.26, 115.53]
**Subtotal (95% CI)**		**190**		**208**	**2.0%**	**7.67 [0.93, 63.59]**
Total events	6		0			
Heterogeneity: Tau² = 0.00; Chi² = 0.09, df = 1 (P = 0.76); I² = 0%						
Test for overall effect: Z = 1.89 (P = 0.06)						
**Total (95% CI)**		**1297**		**1311**	**100.0%**	**1.92 [1.41, 2.61]**
Total events	655		527			
Heterogeneity: Tau² = 0.14; Chi² = 23.37, df = 11 (P = 0.02); I² = 53%						
Test for overall effect: Z = 4.12 (P < 0.0001)						
Test for subgroup differences: Chi² = 1.68, df = 1 (P = 0.19), I² = 40.6%						

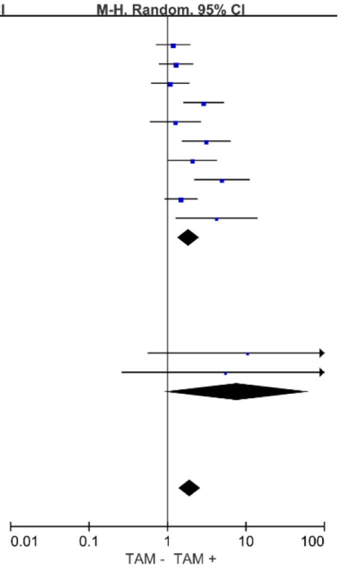

Supplementary Figure S3: Subgroup analysis of OS according to TAM density in different TNM stages of tumor. A. 3-year OS; **B.** 5-year OS. TI: tumor islet; TS: tumor stroma; M1: CD68 and HLA-DR positive cells; M2: CD163 positive cells, CD204 positive cells, CD68 and CD163 positive cells, CD68 and CD206 positive cells or IL-10 and CD68 positive cells.
